# Understanding Cervical Cancer Screening Barriers among Migrant Women: A Qualitative Study with Healthcare and Community Workers in Portugal

**DOI:** 10.3390/ijerph18147248

**Published:** 2021-07-06

**Authors:** Patrícia Marques, Ana Gama, Mário Santos, Bruno Heleno, Heleen Vermandere, Sónia Dias

**Affiliations:** 1 NOVA National School of Public Health, Public Health Research Centre, Universidade NOVA de Lisboa, 1600-560 Lisbon, Portugal; psm.marques@ensp.unl.pt (P.M.); ana.gama@ensp.unl.pt (A.G.); mario.santos@ensp.unl.pt (M.S.); 2 Comprehensive Health Research Centre (CHRC), Universidade NOVA de Lisboa, 1169-056 Lisbon, Portugal; bruno.heleno@nms.unl.pt; 3 NOVA Medical School, Universidade Nova de Lisboa, 1169-056 Lisbon, Portugal; 4 International Centre for Reproductive Health, Ghent University, 9000 Ghent, Belgium; Heleen.Vermandere@UGent.be

**Keywords:** sexual health, cervical cancer screening, migrant women, healthcare workers, community workers

## Abstract

Cervical cancer screening (CCS) has been proven to reducing mortality of cervical cancer; yet migrant women show a lower participation in screening compared to non-migrants. This study explores the perspectives of healthcare workers and community workers on the factors influencing the CCS participation of migrant women living in Portugal. A qualitative study with online focus groups was conducted. Healthcare workers experienced in CCS and community workers working with migrant communities were purposively sampled. A semi-structured guide was used covering the participation of migrant women in CCS, barriers, and strategies to overcome them. Data were analyzed using content analysis. Participants considered that migrant women have low participation in CCS related to insufficient knowledge, low risk perception, and lack of interest on preventive care. Other barriers such as difficulties in accessing the healthcare services, relationship with healthcare workers, language, and cultural differences were highlighted. Promoting continuity of care, disseminating culturally tailored information, and use of self-sampling methods were suggested to improve participation in CCS. Inequalities in access to CCS among migrant women are mostly caused by information gaps and healthcare system-related barriers. Building a migrant-friendly healthcare system that creates opportunities for healthcare workers to establish relationships with their patients and delivering culturally and linguistically adapted information may contribute to overcoming those barriers and increasing the participation of migrant women in screening.

## 1. Introduction

Cervical cancer remains one of the most common cancers among women worldwide, with estimates for 2020 indicating an incidence rate of 17.6 and a mortality rate of 8.5 per 100,000 women aged 15–64 years [1]. In Portugal, incidence and mortality rates of the disease were 15.4 and 4.6, respectively, per 100,000 women aged 15–64 in 2020 [1].

Nearly all cervical cancers result from an infection by oncogenic genotypes of the human papilloma virus (HPV). The disease is highly preventable through HPV vaccination among young adolescents but also through regular screening among women given its long latency period [2,3].

Through cervical cancer screening (CCS), precancerous lesions are detected; treatment of these lesions can stop further progression to cervical cancer [4]. CCS involves the collection of cervical cells usually performed by a clinician during a gynecological examination at a health facility, but self-sampling has been explored as an alternative [4,5]. Evidence shows that CCS reduces morbidity and mortality [6,7]. Therefore, CCS should be easily accessible to all eligible women according to the respective national guidelines [4,6,8].

Despite the efforts to implement screening strategies, inequalities persist. This is particularly true among migrant women, who often have a lower participation in CCS when compared to non-migrant women [9,10]. As evidenced by research in Europe, migrant women present a higher risk of being diagnosed with cervical cancer at a later stage, resulting in increased morbidity and mortality [4,9].

Several factors influencing migrant women’s participation in CCS have been documented in European countries, such as language and communication difficulties, lack of knowledge on screening, negative perceptions and feelings, cultural differences, as well as barriers related to healthcare services and provider–patient relationship [11].

In Portugal, the foreign-born population in 2019 represented 10.8% of the total population residing in the country, and nearly half of these individuals were women, mostly from Brazil, Portuguese-speaking African countries, Eastern Europe, and China, which are countries with a high incidence of cervical cancer [1,12,13].

Portugal has a population-based CCS program that is organized by regional authorities [14]. All women registered at a primary healthcare center are invited to participate in CCS through a letter (written in Portuguese or English), which is usually performed by a general practitioner, every 3 to 5 years, depending on the region [15]. Additionally, CCS tests can be carried out opportunistically [16]. There are several examples of CCS programs in other countries, such as the United Kingdom, Finland, and the Netherlands, where population-based CCS programs are implemented nationwide, women are invited regularly for screening, and the programs are intensely disseminated through the population [16,17]. Data from the Portuguese National Health Survey, in 2014, indicate that migrant women have a lower participation in CCS than women born in Portugal [18].

Overall, it is important to ensure that migrant women access CCS programs in the host countries. According to a recent scoping review, most studies on factors associated with CCS participation among migrant women in Europe focus on women’s perspectives, while a small number of studies have explored the perspectives of healthcare workers, and up to the authors’ knowledge, only an article has included the perspective of a single community worker from a migrant support association [11]. These key stakeholders have privileged inside knowledge on the barriers faced by migrants from a health and social perspective, which can help in the development of strategies to increase CCS participation among migrant women.

This study aims to explore the perspectives of healthcare workers and community workers on the participation of migrant women in CCS in Portugal, by (i) assessing their experiences and opinions about CCS participation of migrant women, (ii) exploring the barriers faced by these women to participate in CCS, and (iii) identifying strategies to overcome these barriers. The findings will contribute to advance knowledge on factors influencing CCS participation among migrant women and can help produce meaningful and culturally competent recommendations to an international audience.

## 2. Materials and Methods

A qualitative study was conducted with healthcare and community workers in Lisbon and Tagus Valley Region using focus groups (FGs), a valuable technique by which participants share their opinions on a certain theme interacting with each other in small groups [19].

General practitioners and public health doctors with experience in CCS from healthcare units in areas with larger migrant communities were invited through e-mail to take part in FGs. Community workers were also invited through contact with associations targeting different migrant populations, to include perspectives about different communities. 

FGs were organized according to the availability of the participants and were planned to include 4 to 8 participants each, with healthcare workers and community workers separately. Discussions were moderated by a member of the research team, experienced in qualitative research.

A semi-structured guide was developed, based on the literature [10,11,20], containing open-ended questions followed by probing questions on topics that covered the participants’ perceptions about migrant women’s participation in CCS, barriers faced by these women, and strategies to overcome them. The semi-structured guide is available in Table S1, in the supplementary file.

FGs were planned to be conducted face to face. However, due to the COVID-19 pandemic outbreak, FGs were conducted through Zoom, an online videoconference platform.

Data were analyzed using a content analysis approach [19]. A codebook with themes, subthemes, and main codes was constructed based on the existing evidence [11,20]. All FG were recorded, transcribed verbatim, and anonymized. Each transcript was analyzed separately, and segments of the text were categorized into codes defined in the codebook, using a word processor. New codes that emerged during the codification process were added to the codebook. Codes were organized in themes using the ecological model as a framework [21]. Illustrative quotes for each code were selected and translated into English.

All participants signed a written informed consent form and gave oral permission to audio record the sessions. Before each FG, participants were ensured of their anonymity, the confidentiality of the data collected and were informed that they could withdrawal at any stage of the study. Approval was obtained from the Ethics Committee for Health of the Regional Health Administration of Lisbon and Tagus Valley (ARSLVT) (Reference: 8431/CES/2019).

## 3. Results

Four FGs were conducted between June and November 2020. Three FGs were carried out with twelve healthcare workers, including general practitioners (GP) and public health doctors (PH) (one FG with six participants and two FGs with three), as well as one FG with five community workers. FGs lasted for about one hour and half each. 

Characteristics of the participants are presented in Table 1. Most participants had extensive experience working with migrant women from different communities. Each community worker represented a different organization.

### 3.1. Migrant Women’s Participation in CCS

Most healthcare workers confirmed that migrant women have lower participation in CCS when compared to non-migrant women. Some healthcare workers observed that migrant women rarely schedule an appointment specifically for CCS; usually, the opportunity arises in the context of family planning or maternal health consultations:

***HW6 (GP)*** *: I feel that many of them [Asian and African migrants] only realize that screening exists when they try to get pregnant or when they are pregnant. That is, they only find out about screening when they have a maternal health consultation, and someone asks them about it.*

Participants discussed that women participate differently in CCS according to their country of birth. Several participants stated, as an example, that migrants from Brazil frequently seek out CCS, as they seem to be more open to preventive care:

***HW4 (GP)*** *: Among migrant women, the only ones I remember actively seeking to be screened are some of the Brazilian women, among whom the culture of preventive medicine is deeply rooted.*

Healthcare workers stated that most migrant women are unaware of CCS or its benefits, and therefore, they do not seek this preventive care. However, participants feel that even those who seem more reluctant to be screened, such as Asian and African women, are open to CCS when they are informed about it. This opinion was shared by community workers:

***HW1 (GP)*** *: I noticed that the African populations are very different [among each other]. (...) But generally they are women who access [healthcare] more when they have complaints, and then they accept and do the screening, but they do not do it spontaneously (…).*

***CW2*** *: I would say that [migrant] women who are followed up in health services, as in primary healthcare centers in general medicine, (…) if they are asked and offered cervical screening, they do not refuse.*

Participants agree that migrant women who have regular contact with healthcare services are likely to accept CCS when it is offered by their doctor.

### 3.2. Barriers to CCS among Migrant Women

Barriers to CCS were grouped in three subthemes: individual, sociocultural, and health system-related barriers (Table 2).

#### 3.2.1. Individual Barriers

- Lack of information about CCS

Nearly all participants stated that most migrant women do not know what cervical cancer is, and do not know what CCS is for, nor where it can be done:

***CW3*** *: I think that among [migrant] women there is a lack of literacy in knowing what cervical cancer is, when can it [screening] be done, from what age, for what…*

Healthcare workers mentioned that migrant women are largely unaware of CCS, which is reflected in low uptake. Some participants mentioned that CCS is a sensitive topic and misinformation about where and how to do it exists. Additionally, CCS information is scarce and may not be sufficient for migrant women to understand the invitation for screening, negatively affecting their participation:

***HW5 (GP)*** *: If I, as a migrant woman, don’t know what I’m going to do [in screening] and why should I do it, what are the benefits [of screening], I’m not doing it.*

- Perceptions about CCS

Women’s perception of low risk for cervical cancer and low impact in their life was another reason mentioned in FGs for not participating in CCS. In addition, according to some community workers, migrant women often do not consider CCS relevant and have a lack of interest in preventive care:

***CW4*** *: (…) the world of an immigrant is so busy most of the times that people don’t even care about it [screening] (…). It is important for people to know that they may not have much time, but they must find the time to take care of their own health (…)*

- Older age

Participants mentioned that older migrant women usually are not so open to participate in CCS. During the discussions, they stated that, as women get older, they tend to talk less about their sexual and reproductive health and avoid gynecological exams, such as CCS. Community workers referred that older women believe they no longer need to be screened, as they are over their reproductive period:

***CW3*** *: (…) considering the experience I have with [migrant] people, older women, those who already have children, who have already “closed the store”, so to speak, don’t want to do the cervical test, they don’t care about it.*

#### 3.2.2. Sociocultural Barriers

- Language and communication difficulties

Language barriers affect effective communication, and this was one of the main barriers discussed in all FG. Participants said that explaining the purpose of CCS may be challenging, as it is an intimate exam. Despite the effort to provide information in English, healthcare workers stressed that many women coming from non-Portuguese speaking countries do not speak English either:

***HW1 (GP)*** *: I think the language barrier is a very, very important barrier among migrant populations. Although we use English, not all migrant populations use that language well or know the language.*

- Religion and culture-related barriers

In all FG, participants reported that culture influences attitudes regarding preventive health and helps to explain prejudice, fear, or discomfort towards gynecological examination:

***HW3 (GP)*** *: I think that the cultural part turns out to be essential, and while we have already started to have some notion of prevention, there are many cultures dealing with the “now”, and everything that is prevention does not exist.*

***HW1 (GP)*** *: I feel that some [migrant] women are afraid of doing the exam (…)*

Religion also influences women access to CCS. According to the experience of healthcare workers, Muslim women seem to have greater difficulty in taking up CCS, as they tend to be more reserved and less autonomous:

***HW10 (GP)*** *: The religious part [of our migrant communities], (…), the Muslim part, the part that is more influenced by Islam ends up diminishing the autonomy of women.*

Indeed, religious and cultural background also influences gender roles and relations, affecting uptake of CCS. Participants reported that some aspects of women’s health are managed by their husbands, who usually have a better understanding of the language and are often present in their wives’ appointment, deciding if CCS is performed. This can raise problems in terms of privacy and self-determination and can hamper women from sharing their concerns with the practitioner. Additionally, some participants reported situations where the husband did not allow his wife to have a gynecological exam or to be seen by a male doctor, even if she felt comfortable doing so, particularly among Muslim communities:

***HW6 (GP)*** *: It is obvious that there are men, because of this language barrier, who do not even let their wife be observed by a man, right? And it is not the wife herself who says she doesn’t want it, it’s the husband who doesn’t allow it. And since he has the power to manage the access [of his wife] to the consultation because he is the one who usually speaks the language, it ends up being an indirect barrier here.*

Some community workers stated that the migrant women’s family can also influence their participation by inducing myths and prejudices about the gynecological exam. On the other hand, a healthcare worker pointed out that a migrant woman who has never been screened may want, in the consultation, the company of a trusted family member who has experienced CCS:

***HW2 (GP)*** *: (…) many of the migrant women, or those who have never been screened, will want to be screened when they are accompanied in consultation by a female friend or family member, who has already done it and who they trust.*

Participants also identified that women coming from countries with poorer healthcare access may avoid attending healthcare services in the host country, which in turn negatively impacts their CCS participation:

***HW12 (PH)*** *: If we are talking about a migrant community coming from countries where access to healthcare is poorer, (…) we will eventually be talking about people who are, in fact, ideologically difficult to access, in terms of healthcare and specifically of screening.*

- Female genital mutilation

Some healthcare workers reported that practices of female genital mutilation frequently result in shame and pain, leading these migrant women to avoid CCS:

***HW3 (GP)*** *: I would say that female genital mutilation and screening is something that doesn’t connect well, right? Therefore, these women try at all costs to hide from the gynecological exam, and everything that has to do with it. And sometimes it’s even impossible as they are sutured...*

#### 3.2.3. Health System-Related Barriers

- Procedural difficulties in accessing the healthcare services

Healthcare workers stated that in general migrant women have greater difficulties in accessing health services compared to non-migrant women, which negatively affects the access to CCS:

***HW4 (GP)*** *: [A barrier is] access to healthcare, regardless of everything else (…) Because to be able to access the screening program one must navigate a series of bureaucratic procedures until being able to access the National Health Service (NHS), in terms of some groups of migrants (…).*

It was also specified that not having an NHS user number nor having an assigned family doctor can negatively influence access to health services and participation in CCS:

***HW1 (GP)*** *: I have already had situations in which patients did not [do the screening] because, as the prescription is handwritten and there is no NHS user number, the test requisition doesn’t come out of the program.*

***CW2*** *: In [city] the problem is that many [migrant women] do not have a family doctor, so they are not followed, at least regularly, in health centers [therefore screening is not proposed].*

Healthcare workers acknowledged that the information made available, mainly through invitation letters and leaflets in healthcare facilities, is not informative enough nor adapted to the cultural and linguistic diversity of migrant populations. Thus, migrant women often are unable to understand the importance and benefits of CCS through the letter they receive:

***HW10 (GP)*** *: Even so, there are many [migrants] who do not understand, they receive the letter [invitation for screening], they realize that it may be to go [to the healthcare center] or what they have to do, but it is not enough for them to be aware, for example, of the risk.*

- Healthcare workers characteristics and relationship with patients

Participants in all FG pointed out that some women, particularly Muslim women, are frequently uncomfortable or refuse to be examined by male doctors. Community workers also pointed out that some characteristics of healthcare workers, such as the age and country of origin, can influence participation in CCS:

***HW2 (GP*** *): I think women who are Muslim tend to be more reserved and I assume that I will not be able to do the screening myself, as a male doctor. So, when I propose the screening, I’m aware that probably there will be this cultural barrier, and I often say that it is possible to have the exam done by a woman, if she wishes so. And only then something [screening] can be achieved. Sometimes Muslim women do not mind being screened by a man, but it is very rare. Very rare.*

***CW1*** *: It is not only the gender, but also the origin of the doctor, and the age, because for older women it is very difficult to be observed by younger women. It has an enormous cultural weight to expose your femininity to a doctor of a younger age.*

Healthcare workers assumed that there is a lack of representativeness of the migrant community in the health sector, which hinders the doctor–patient relationship and often negatively influences the acceptability of CCS when proposed:

***HW2 (GP)*** *: I think that in terms of healthcare teams there is a huge gap. Teams do not represent their own community, that is, we do not have nurses or doctors from that community, so sometimes there is no cultural connection or cultural empathy that patients could often feel if, for example, the health professional who proposed the test or talked about the test was from the same culture or background.*

Some healthcare workers emphasized that the doctor–patient relationship influences CCS participation in that, if a migrant woman does not trust the doctor, she will not be willing to undergo CCS. Healthcare workers also mentioned that the time allocated for the consultation is seldom enough to establish a relationship with the patient, and often, they meet the patient only once, which hampers the establishment of a trusting bond. Participants found this issue to be relevant for CCS because it is an intimate test, and women may feel uncomfortable exposing themselves to someone they do not know, which reduces participation:

***HW1 (GP)*** *: But here, where we see a patient sometimes only once, and we don’t see her again, introducing this concept [of screening] ... there is no time to establish trust, or time to establish the relationship, and people don’t want to expose an intimate part .... they don’t want to do it and, therefore, it’s also a difficulty.*

- Irregular status

The irregular status of migrant women was a barrier discussed in all groups, but particularly among community workers. They pointed out that undocumented women face additional obstacles in accessing healthcare services and are not covered by the CCS program:

***CW3*** *: For migrant women, there are several issues [for not being tested]. The fact that they are not legalized and therefore do not have access to the NHS for free.*

- Outdated patient information

Participants mentioned that migrant women often change their address or their telephone number, mostly due to economic difficulties:

***CW2*** *: Another issue with the phone that we encounter frequently is that the people we work with are also people with severe economic needs; people change their phone number every month (…) because they buy that card that is cheaper and at the end of the month that card no longer has calls available, and they buy another one.*

For this reason, some healthcare workers reported that the databases with patients’ information and contacts are constantly outdated, which precludes migrant women from receiving invitation calls or letters for CCS.

### 3.3. Improving Migrant Women’s Participation in CCS

Strategies to overcome the barriers identified were gathered into three groups—continuity of care, culturally tailored information, and self-sampling methods. 

#### 3.3.1. Continuity of Care

Health professionals suggested investing in continuity of care and building a strong doctor–patient relationship as one of the possible strategies to increase participation in CCS among migrant women. This close relationship would allow healthcare workers to help promote the health literacy of these women, and consequently, they would be more willing to participate in health-related interventions, including CCS:

***HW5 (GP)*** *: In other words, from my point of view, one of the solutions [to increase adherence] is (…) continuity of care.*

***HW10 (GP)*** *: (…) And if she has some kind of relationship with healthcare services, she is also much more receptive to be targeted by health education and to improve her own health literacy, and thereby change her risk perception or, as someone here also said, change the reason why she will want to do the screening.*

However, as healthcare workers mentioned, to establish these relationships it is necessary that health services are prepared to receive migrant women, considering their culture, language, and specific needs. The time allocated to consultations should be increased, and women should always have the possibility of choosing a doctor they feel comfortable with:

***HW12 (PH)*** *: [Health services] must be prepared to receive these populations, not only in terms of language, but also in specificities of their cultures, their traditions.*

***HW11 (GP)*** *: And then the question of the time that is allocated, or the availability of the professional, or the question of whether or not you are a family doctor, so here too the attention that is given to details is important and it is the best occasion [to pass on information].*

Participants generally agreed that having an assigned family doctor for each migrant woman would help improve their access to CCS:

***HW10 (GP)*** *: If we manage to have (…) for example, family doctors for everyone in national terms, 100% coverage, I think, not all, but an important number of barriers would disappear.*

***CW2*** *: It is in the consultation itself that it [CCS] can be done or that it can be scheduled, but with the same doctor. So here it can facilitate [access to CCS] if we have a good relationship with our family doctor; it can be a facilitator, because [the doctor] is the one who will do it.*

#### 3.3.2. Culturally Tailored Information

Some participants reported that, to increase participation in CCS, migrant women need to have access to appropriate and culturally adapted information about the benefits of CCS. Participants mentioned that information campaigns targeting specific migrant communities, taking different cultural, educational, and linguistic contexts into account, would be the best strategy to deliver information. The support of community associations or community leaders to deliver information about CCS was discussed by the participants and generally accepted as a good approach:

***CW3*** *: It is necessary that we go to the field and make connections with entities that deal with this population. We are talking perhaps about the National Immigrant Support Centers, the immigrants’ associations, for example, but at the core—I mean in the neighborhoods—where people live, to adapt and direct communication to this audience.*

#### 3.3.3. Self-Testing Methods

For some participants, the use of self-sampling CCS tests is a good alternative to minimize some barriers related to the discomfort, fear, and shame associated with the test:

***HW5 (GP)*** *: A solution could be a self-sampling test (…). Such as the ones done for the fecal occult blood test, or even in some countries when screening for chlamydia. If I send someone a bottle to self-collect, it is much more likely that the person will participate.*

***CW4*** *: I believe that the advantage of self-sampling is important and if this possibility is put in place, I believe that [it may help overcoming barriers], in the practical sense of analysis and examination and of people’s own safety, because many women are still afraid and have prejudice of consulting gynecologist doctors, right?*

## 4. Discussion

This qualitative study explored the perspectives of healthcare workers and community workers regarding the experiences, barriers, and strategies to increase participation in CCS among migrant women in Portugal. 

Participants agreed that migrant women have a lower participation in CCS comparing to non-migrant women, confirming other studies [18,22,23,24]. Previous experiences with preventive healthcare may increase CCS participation [11,20,25]. Healthcare workers commented that women coming from countries where preventive healthcare is promoted, such as Brazil, seem to be more proactive when it comes to CCS. Migrant women with lower knowledge and awareness about preventive measures tend to only participate in CCS opportunistically when seeking for healthcare services for other reasons [11,20]. Some participants, mainly community workers, highlighted that if a healthcare worker takes the initiative to explain what CCS is and why it is important, migrant women are open to participate.

Among the barriers identified, lack of information, language and communication difficulties, and structural obstacles in access to healthcare services were the most discussed factors among healthcare and community workers. These barriers are in line with those found in other studies [11,20]. When discussing barriers, healthcare workers focused more on the organization of health services, while community workers highlighted sociocultural and legal issues faced by migrant women when accessing CCS.

Overall, it was highlighted that several of the barriers that migrant women face to access CCS are related to difficulties in accessing healthcare services in general, which has also been observed in other countries [9,10]. Healthcare services often lack cultural diversity within their staff and skills to deal with cultural and linguistic differences and frequently display complex bureaucratic processes for access, which can be challenging for migrants [11,25,26]. Participants also noted that certain characteristics of the healthcare worker, such as sex, age, or cultural background, may lead some migrant women to refuse CCS. On the other hand, irregular migration status also emerged in FGs as a barrier to CCS. Community workers referred that women in irregular situations still face barriers to access healthcare services and, consequently, to CCS. Healthcare workers also mentioned that migrant women frequently change their address and phone number due to economic constraints, and this unstable living situation becomes a major challenge given that, in Portugal, invitations for CCS are made by post mail or phone call [15]. 

Some cultural aspects were identified during the discussion to a lesser extent, with the role of the husband or partner being the most discussed. As documented elsewhere [25,26], men generally act as translators in consultations, and often, they have the power to decide if their wife participates in CCS. Healthcare workers expressed some frustration, as they felt that they had to persuade the husband to let his wife be screened for cervical cancer, which raised the issue of the lack of autonomy of some women in managing their own health.

Although most of the migrants residing in Portugal are from Portuguese-speaking countries [12], communication and language issues were considered relevant. Information transmission is an important issue [25,27,28,29]; participants felt that the written information available was too complex, which comprises a major challenge for women who are not fluent in Portuguese and who have low health literacy. Additionally, participants stated that if women are unable to understand the benefits of CCS, they are less willing to participate. Knowledge acquisition is strongly related to how the message is transmitted [26,27,30], and based on the participants experience, the way information is provided may not be adapted to the cultural and linguistic context of these communities, resulting in a lack of knowledge about CCS. 

Within an ecological perspective, efforts to increase individuals’ awareness and up-take of CCS can only be effective if they address changes in the social environment structure and processes [21]. The perspectives of healthcare and community workers, which have not been much explored in research, are helpful to design better strategies to increase CCS participation among migrant women, especially those groups who are not being reached by CCS programs. These professionals work closely with migrant communities have deep knowledge of these women’s difficulties when accessing healthcare services and the barriers related to preventive care and women’s health. These professionals can provide valuable insights on who are the groups with low participation in CCS, which factors influence their participation, and how to better reach them and improve their uptake of CCS. 

At system level, strategies must be developed to reach migrant women who are not participating in the CCS program, particularly women living in unstable conditions due to economic constraints and undocumented women. This may be achieved by using different invitation methods other than letters, providing opportunities for these women to seek CCS in proximity healthcare units, and creating information campaigns aimed at these specific groups. For example, in the United Kingdom, the National Health System promotes events and uses the media, namely national TV and social media, to increase awareness about CCS program [16,17].

Building a trusting doctor–patient relationship in a migrant-friendly healthcare system that takes into consideration migrant women’s specific cultural needs seems to be key to improve their participation in CCS [11,25,26], as explored in the FGs. Strengthening the skills and cultural competences of healthcare workers to deal with populations from different sociocultural backgrounds and increasing the duration of medical appointments may be relevant to build a trusting bond between these professionals and the patients, and to provide information about CCS more effectively. Establishing partnerships with community associations, as suggested by the participants, may also help delivering culturally and linguistically adapted information targeting these populations [20,25,26,28]. Community workers who work closely with migrant communities have a deeper knowledge on the difficulties faced by these women, often share language and cultural backgrounds with them, and can help to reach those who are not being screened making them key actors to improve CCS uptake among migrant women.

A strategy suggested by the participants to help reduce barriers to CCS is offering the possibility of using self-sampling tests. This has been explored as a potentially good alternative to conventional CCS that can increase participation among migrants [5].

This study has some limitations. Data report the views of a small number of participants, due to the pandemic outbreak and the consequent difficulty in recruiting healthcare workers and community workers whose services have been overwhelmed in this crisis situation. Nevertheless, information saturation was reached in FGs with healthcare workers. Participants worked only in the Lisbon and Tagus Valley Region; notwithstanding, the largest migrant communities are located in this region [13]. Additionally, there is an imbalance in sex distribution, with most participants being female. Nevertheless, this reflects the features of the healthcare and community workers reported worldwide and in Portugal as well [31,32,33]. Furthermore, the virtual setting partially limited the group dynamics, making it challenging to assess non-verbal cues.

## 5. Conclusions

The findings of this study bring to light some of the possible factors acting as barriers to participation of migrant women in CCS initiatives that can be meaningful for an international audience, particularly in the European context. The inclusion of healthcare and community workers gave a broader social perspective on inequities in access to CCS and offers a new, experience-based view on who is not being included in the CCS program, how to reduce the barriers, and increase the participation of these migrant groups. Participants pointed out that the inequalities in accessing CCS among some migrant women are most likely caused by a lack of information, language and communication difficulties, and obstacles in accessing healthcare services. Developing a migrant-friendly healthcare system with culturally tailored information and creating opportunities for healthcare workers to establish trusting relationships with patients seems to be crucial to improve migrant women’s participation in CCS. Specific attention should be given to women living in unstable conditions due to economic constraints, undocumented women, and those without a family doctor, to include them in CCS program. Additionally, establishing partnerships with community associations that are close to migrant communities to deliver information and improve awareness of CCS may contribute to overcoming some of the identified barriers.

## Figures and Tables

**Table 1:** Characteristics of the participants.

	Healthcare Workers (*n* = 12)	Community Workers (*n* = 5)
Age		
<35 years	5	3
≥35 years	7	2
Sex		
Male	3	1
Female	9	4
Migration background		
Migrant	1	4
Non-migrant	11	1
Medical speciality		
General Practitioner	10	-
Public Health Doctor	2	-
Years of medical practice		
≤5 years	3	-
>5 years	9	-
Experience working with CCS		
≤5 years	4	-
>5 years	8	-

**Table 2:** Barriers to CCS described in FGs.

Barriers to CCS		
Individual	Sociocultural	Health System-Related
Lack of information about CCS Perceptions about CCS Older age	Language and communication difficulties Religion and culture-related barriers Female genital mutilation	Procedural difficulties in accessing the healthcare services Healthcare workers characteristics and relationship with patients Irregular status Outdated patient information

## Data Availability

The data presented in this study are available on reasonable request from the corresponding author. The data are not publicly available due to privacy reasons.

## Supplementary Materials

The following are available online at https://www.mdpi.com/article/10.3390/ijerph18147248/s1, Table S1: Semi-structured guide for the focus groups.
