# Supplementary material for: Understanding Cervical Cancer Screening Barriers among Migrant Women: A Qualitative Study with Healthcare and Community Workers in Portugal
Source: Int J Environ Res Public Health. 2021 Jul 6;18(14):7248. doi: 10.3390/ijerph18147248 (PMC8305801; doi:10.3390/ijerph18147248)
Supplement: Supplementary file 1 [file ijerph-18-07248-s001.zip › ijerph-1250249-supplementary.pdf]

Table S1. Semi-structured guide for the focus groups.

**Perceptions about migrant women's participation in CCS**
• What are your opinions about migrant women's participation in cervical cancer screening? What about their participation compared with the general population?<br>• How would you describe the women who have a lower participation in CCS?
**Barriers to CCS participation among migrant women**
• What do you think are the main factors influencing migrant women's participation in CCS?<br>• From your experience, what are the barriers faced by migrant women to CCS?<br>• What about individual barriers to CCS faced by migrant women? And sociocultural barriers? What can you tell about health system-related barriers?
**Strategies to overcome barriers to CCS among migrant women**
• How could these barriers be addressed?<br>• In your opinion, what kind of strategies could help reduce the barriers?
